# Alterations of the thalamic nuclei volumes and intrinsic thalamic network in patients with restless legs syndrome

**DOI:** 10.1038/s41598-023-31606-8

**Published:** 2023-03-17

**Authors:** Kang Min Park, Keun Tae Kim, Dong Ah Lee, Yong Won Cho

**Affiliations:** 1 Department of Neurology, Haeundae Paik Hospital, Inje University College of Medicine, Busan, Korea; 2 Department of Neurology, Keimyung University School of Medicine, 1035 Dalgubeoldae-ro, Dalseo-gu, Daegu, 42601 Korea

**Keywords:** Neurological disorders, Pathogenesis

## Abstract

We aimed to investigate the alterations of thalamic nuclei volumes and intrinsic thalamic network in patients with primary restless legs syndrome (RLS) compared to healthy controls. Seventy-one patients with primary RLS and 55 healthy controls were recruited. They underwent brain MRI using a three-tesla MRI scanner, including three-dimensional T1-weighted images. The intrinsic thalamic network was determined using graph theoretical analysis. The right and left whole thalamic volumes, and the right pulvinar inferior, left ventral posterolateral, left medial ventral, and left pulvinar inferior nuclei volumes in the patients with RLS were lower than those in healthy controls (0.433 vs. 0.447%, *p* = 0.034; 0.482 vs. 0.502%, *p* = 0.016; 0.013 vs. 0.015%, *p* = 0.031; 0.062 vs. 0.065%, *p* = 0.035; 0.001 vs. 0.001%, *p* = 0.034; 0.018 vs. 0.020%, *p* = 0.043; respectively). There was also a difference in the intrinsic thalamic network between the groups. The assortative coefficient in patients with RLS was higher than that in healthy controls (0.0318 vs. − 0.0358, *p* = 0.048). We demonstrated the alterations of thalamic nuclei volumes and intrinsic thalamic network in patients with RLS compared to healthy controls. These changes might be related to RLS pathophysiology and suggest the pivotal role of the thalamus in RLS symptoms.

## Introduction

Restless legs syndrome (RLS) is the most significant of the sleep related movement disorders, causing significant health and personal morbidity, including chronic sleep deprivation, depression, and anxiety^1,2^. In studies, RLS of varying severity affects between 3.9% and 15% of adults, and females have a higher rate than males^2–5^. However, RLS has been not widely recognized, diagnosed, and treated.

Although the exact pathogenesis for RLS has not been clearly demonstrated, there have been many studies and various causes have been suggested. Among them, reduced regional brain iron stores^6–8^ and dopaminergic system dysfunction^9,10^ are the most consistently implicated central nervous system abnormalities. Lower peripheral iron status has been associated with an increased risk of developing and worsening RLS symptoms, and both brain magnetic resonance imaging (MRI) and ultrasound imaging studies have revealed iron deficiency in the region of the substantia nigra in patients with RLS^6–8^. The observation that dopaminergic therapy improves RLS symptoms strongly implicates the pivotal role of dopamine in the pathogenesis of RLS, and the dopamine transporter is decreased in the striatum of patients with RLS, demonstrated by positron emission tomography studies^9,10^.

RLS is defined by sensory symptoms, not movement, and is based on the essential characteristic of a frequently unpleasant or inconvenient urge to move the legs that occurs during periods of inactivity, particularly in the evenings, and is temporarily alleviated by movement^1,2^. Because the main features of RLS are sensory symptoms, we focused on the thalamus in this study, which is the most important sensory-associated structure in the brain. Previous studies have also frequently reported thalamic abnormalities in patients with RLS^11^. A study using proton magnetic resonance spectroscopy has demonstrated decreased metabolite activity in the medial thalamus with decreased N-acetyl aspartate to creatine ratio^12^. In a small pathologic study, the number of beta-endorphin and met-enkephalin positive cells in the thalamus was reduced in patients with RLS compared to healthy controls^13^. These studies suggest the association between the thalamus and RLS pathophysiology.

Recently, neuroimaging has been widely used to investigate the pathophysiologic mechanisms for various neurological diseases, with breakthrough developments of techniques for MRI scanning and analysis. A new technical tool generates a parcellation of the thalamus into 25 distinct nuclei using a probabilistic atlas constructed from histological data^14^. By utilizing this tool, it is possible to measure each individual thalamic nuclei volume, as well as the total volume of the thalamus. These MRI-based thalamic nuclei volume analysis has demonstrated a good agreement with histological findings and showed an excellent test–retest reliability^14^. The various thalamic nuclei have a distinct location and function, and each thalamic nuclei is connected differently to a specific brain regions. In addition, a graph theoretical analysis based on these thalamic nuclei volumes allows the calculation of the intrinsic thalamic network in subjects^15–17^. Graph theory metrics can provide a valuable assessments and sophisticated characterization of the intrinsic thalamic network. Thus, structural co-variance analysis based on the thalamic nuclei volumes with a graph theory could conceptualize how morphological properties of different thalamic nuclei relate to each other at the group level, which could be related with RLS pathophysiology. However, there have been no studies to investigate the change of thalamic nuclei volumes or intrinsic thalamic network in patients with RLS compared to healthy controls.

In this study, we aimed to investigate the changes in thalamic nuclei volumes and the intrinsic thalamic network in patients with primary RLS compared to healthy controls. We hypothesized that there were significant changes in the thalamic nuclei volumes and intrinsic thalamic network in patients with RLS.

## Results

### Demographic and clinical characteristics

Table 1 reveals the demographic and clinical characteristics in patients with RLS and healthy controls. The ages and sex were not significantly different (age, 56.4 vs. 55.4 years, *p* = 0.415; males, 18/71 (25%) vs. females, 17/55 (30%), *p* = 0.491; respectively). However, the Pittsburgh sleep quality index, Insomnia severity index, Hospital anxiety scale, and Hospital depression scale were higher in patients with RLS than healthy controls (12 vs. 4, *p* < 0.001; 17 vs. 3, *p* < 0.001; 7 vs. 4, *p* < 0.001; 8 vs. 5, *p* = 0.001; respectively). Of the 71 patients with RLS, 21 patients (29.5%) were already taking medication to relieve RLS symptoms prior to MRI scanning (drug-treated state), whereas the 50 patients (70.5%) were drug-naïve state for RLS.

**Table 1:** Demographic and clinical characteristics in patients with restless legs syndrome.

	Patients with RLS (N = 71)	RLS Patients with drug-naïve state (N = 50)	RLS Patients with drug-treated state (N = 21)	Healthy controls (N = 55)	*p* value*	*p* value^†^	*p* value^‡^
Age, years	56.4 ± 6.5	54.1 ± 5.6	62.1 ± 4.7	55.4 ± 7.2	0.415	0.307	< 0.001
Male, n (%)	18 (25.7)	12 (24.0)	6 (28.5)	17 (30.9)	0.491	0.431	0.843
Age of onset, years	47 (40.2–53.7)	45 (38.0–49.0)	54 (48.2–55.5)				
Symptom duration, months	120 (40–180)	120 (42–180)	120 (36–195)				
RLS severity	26.5 ± 6.7	26.6 ± 7.2	26.4 ± 5.6				
Disease-specific quality of life	8.3 ± 3.7	8.3 ± 3.9	8.4 ± 3.1				
PSQI	12 (9–14)	11.5 (8–14)	13 (9–15.2)	4 (3–5)	< 0.001	< 0.001	< 0.001
ISI	17 (11–23)	16 (11–23)	19 (9.7–22.2)	3 (1–4)	< 0.001	< 0.001	< 0.001
HAS	7 (4–9)	7 (3.8–9)	7 (5–10)	4 (2–5)	< 0.001	< 0.001	< 0.001
HDS	8 (5–10)	8 (4.8–11)	8 (5–9)	5 (4–7)	0.001	0.008	0.004

*RLS* Restless legs syndrome, *PSQI* Pittsburgh sleep quality index, *ISI* Insomnia severity index, *HAS* Hospital anxiety scale, *HDS* Hospital depression scale.

*p value between patients with RLS and healthy controls.

^†^*p* value between RLS patients with drug-naïve state and healthy controls.

^‡^*p* value between RLS patients with drug-treated state and healthy controls.

### Whole thalamic volumes and individual thalamic nuclei volumes

Table 2 shows the difference of whole thalamic volumes and individual thalamic nuclei volumes between patients with RLS and healthy controls. There were significant differences in whole thalamic volumes and some thalamic nuclei volumes between the groups. The right and left whole thalamic volumes in the patients with RLS were lower than those in healthy controls (0.433 vs. 0.447%, *p* = 0.034; 0.482 vs. 0.502%, *p* = 0.016; respectively). Furthermore, the right pulvinar inferior, left ventral posterolateral, left medial ventral, and left pulvinar inferior nuclei volumes in the patients with RLS were lower than those in healthy controls (0.013 vs. 0.015%, *p* = 0.031; 0.062 vs. 0.065%, *p* = 0.035; 0.001 vs. 0.001%, *p* = 0.034; 0.018 vs. 0.020%, *p* = 0.043; respectively).

**Table 2:** Differences in thalamic nuclei volumes between patients with restless legs syndrome and healthy controls.

		Patients with RLS (N = 71)		RLS patients with drug-naïve state (N = 50)		RLS patients with drug-treated state (N = 21)		Healthy controls (N = 55)		Adjusted* p* value*	Adjusted* p* value^†^	Adjusted* p* value^‡^
		Mean (%)	SD (%)	Mean (%)	SD (%)	Mean (%)	SD (%)	Mean (%)	SD (%)			
Rt. whole thalamus		0.4331	0.0350	0.4313	0.0359	0.4370	0.0333	0.4476	0.0420	0.034	0.006	0.316
Lt. whole thalamus		0.4820	0.0427	0.4789	0.0418	0.4890	0.0450	0.5026	0.0409	0.016	0.034	0.316
Right thalamic group	Nucleus											
Anterior	Anteroventral	0.0111	0.0015	0.0108	0.0014	0.0118	0.0014	0.0112	0.0016	0.815	0.297	0.525
Lateral	Laterodorsal	0.0027	0.0007	0.0027	0.0006	0.0028	0.0008	0.0028	0.0008	0.798	0.670	0.913
	Lateral posterior	0.0089	0.0013	0.0086	0.0012	0.0094	0.0015	0.0092	0.0015	0.244	0.097	0.888
Ventral	Ventral anterior	0.0301	0.0028	0.0300	0.0028	0.0304	0.0028	0.0303	0.0036	0.847	0.678	0.913
	Ventral anterior magnocellular	0.0023	0.0002	0.0023	0.0002	0.0023	0.0002	0.0023	0.0002	0.503	0.498	0.888
	Ventral lateral anterior	0.0412	0.0036	0.0411	0.0037	0.0414	0.0034	0.0416	0.0037	0.645	0.604	0.913
	Ventral lateral posterior	0.0531	0.0046	0.0530	0.0048	0.0532	0.0042	0.0537	0.0046	0.491	0.547	0.888
	Ventral posterolateral	0.0517	0.0056	0.0516	0.0052	0.0521	0.0067	0.0531	0.0055	0.310	0.276	0.888
	Ventromedial	0.0013	0.0002	0.0013	0.0002	0.0013	0.0002	0.0014	0.0002	0.245	0.159	0.888
Intralaminar	Central medial	0.0050	0.0006	0.0049	0.0006	0.0050	0.0006	0.0050	0.0006	0.877	0.670	0.906
	Central lateral	0.0033	0.0008	0.0033	0.0008	0.0035	0.0007	0.0034	0.0007	0.798	0.622	0.888
	Paracentral	0.0003	0.0000	0.0003	0.0000	0.0003	0.0000	0.0003	0.0000	0.345	0.648	0.342
	Centromedian	0.0157	0.0017	0.0157	0.0018	0.0157	0.0013	0.0162	0.0017	0.287	0.314	0.724
	Parafascicular	0.0040	0.0004	0.0040	0.0004	0.0039	0.0004	0.0041	0.0004	0.245	0.284	0.525
Medial	Paratenial	0.0005	0.0001	0.0005	0.0001	0.0005	0.0001	0.0005	0.0001	0.441	0.316	0.906
	Medial ventral	0.0011	0.0002	0.0011	0.0002	0.0011	0.0002	0.0011	0.0002	0.873	0.709	0.913
	Mediodorsal medial magnocellular	0.0507	0.0058	0.0512	0.0059	0.0495	0.0052	0.0536	0.0072	0.071	0.179	0.342
	Mediodorsal lateral parvocellular	0.0193	0.0026	0.0194	0.0026	0.0190	0.0028	0.0201	0.0036	0.241	0.365	0.647
Posterior	Lateral geniculate	0.0164	0.0022	0.0166	0.0022	0.0161	0.0024	0.0174	0.0026	0.101	0.149	0.342
	Medial geniculate	0.0083	0.0011	0.0082	0.0010	0.0087	0.0012	0.0087	0.0011	0.188	0.071	0.913
	Suprageniculate	0.0018	0.0004	0.0018	0.0003	0.0018	0.0005	0.0019	0.0004	0.248	0.105	0.888
	Pulvinar anterior	0.0124	0.0015	0.0124	0.0016	0.0125	0.0012	0.0130	0.0015	0.097	0.131	0.525
	Pulvinar medial	0.0666	0.0082	0.0658	0.0085	0.0685	0.0074	0.0704	0.0085	0.097	0.031	0.813
	Pulvinar lateral	0.0115	0.0014	0.0161	0.0022	0.0119	0.0011	0.0113	0.0013	0.457	0.941	0.514
	Pulvinar inferior	0.0139	0.0019	0.0137	0.0019	0.0145	0.0019	0.0150	0.0023	0.031	0.012	0.813
Left thalamic group	Nucleus											
Anterior	Anteroventral	0.0108	0.0011	0.0105	0.0011	0.0113	0.0009	0.0106	0.0013	0.574	0.814	0.342
Lateral	Laterodorsal	0.0025	0.0006	0.0024	0.0005	0.0027	0.0007	0.0026	0.0006	0.279	0.104	0.888
	Lateral posterior	0.0093	0.0014	0.0090	0.0012	0.0099	0.0015	0.0099	0.0013	0.087	0.014	0.913
Ventral	Ventral anterior	0.0308	0.0029	0.0307	0.0030	0.0311	0.0028	0.0311	0.0031	0.814	0.601	0.945
	Ventral anterior magnocellular	0.0025	0.0004	0.0024	0.0003	0.0025	0.0004	0.0025	0.0003	0.401	0.314	0.906
	Ventral lateral anterior	0.0457	0.0050	0.0454	0.0048	0.0465	0.0055	0.0471	0.0045	0.374	0.164	0.888
	Ventral lateral posterior	0.0592	0.0065	0.0587	0.0062	0.0603	0.0070	0.0611	0.0059	0.245	0.164	0.888
	Ventral posterolateral	0.0622	0.0069	0.0617	0.0064	0.0632	0.0081	0.0658	0.0065	0.035	0.012	0.530
	Ventromedial	0.0016	0.0002	0.0015	0.0002	0.0016	0.0003	0.0017	0.0002	0.121	0.010	0.514
Intralaminar	Central medial	0.0054	0.0007	0.0054	0.0007	0.0055	0.0008	0.0056	0.0007	0.245	0.151	0.888
	Central lateral	0.0033	0.0008	0.0031	0.0008	0.0036	0.0006	0.0033	0.0006	0.877	0.410	0.383
	Paracentral	0.0003	0.0000	0.0003	0.0000	0.0003	0.0000	0.0003	0.0000	0.543	0.597	0.888
	Centromedian	0.0172	0.0021	0.0171	0.0023	0.0176	0.0016	0.0178	0.0019	0.245	0.154	0.888
	Parafascicular	0.0039	0.0005	0.0039	0.0005	0.0040	0.0003	0.0041	0.0005	0.139	0.154	0.613
Medial	Paratenial	0.0005	0.0001	0.0005	0.0001	0.0006	0.0001	0.0006	0.0001	0.541	0.301	0.888
	Medial ventral	0.0011	0.0002	0.0011	0.0002	0.0012	0.0002	0.0012	0.0002	0.034	0.012	0.813
	Mediodorsal medial magnocellular	0.0490	0.0058	0.0493	0.0057	0.0482	0.0061	0.0512	0.0052	0.079	0.162	0.342
	Mediodorsal lateral parvocellular	0.0176	0.0022	0.0177	0.0023	0.0174	0.0019	0.0184	0.0019	0.113	0.174	0.383
Posterior	Lateral geniculate	0.0194	0.0029	0.0196	0.0027	0.0189	0.0034	0.0206	0.0029	0.081	0.163	0.342
	Medial geniculate	0.0080	0.0011	0.0078	0.0011	0.0085	0.0010	0.0085	0.0011	0.087	0.016	0.913
	Suprageniculate	0.0017	0.0004	0.0016	0.0003	0.0017	0.0004	0.0017	0.0004	0.714	0.514	0.888
	Pulvinar anterior	0.0152	0.0016	0.0151	0.0015	0.0154	0.0018	0.0159	0.0019	0.087	0.081	0.813
	Pulvinar medial	0.0801	0.0086	0.0792	0.0078	0.0821	0.0102	0.0850	0.0091	0.059	0.014	0.647
	Pulvinar lateral	0.0162	0.0021	0.0113	0.0014	0.0165	0.0020	0.0161	0.0020	0.789	0.943	0.813
	Pulvinar inferior	0.0188	0.0026	0.0187	0.0026	0.0189	0.0025	0.0203	0.0028	0.043	0.032	0.383

*RLS* Restless legs syndrome.

*Adjusted *p* value between patients with RLS and healthy controls.

^†^Adjusted* p* value between RLS patients with drug-naïve state and healthy controls.

^‡^Adjusted* p* value between RLS patients with drug-treated state and healthy controls.

In the comparison of the RLS patients with drug-naïve state and healthy controls, the right and left whole thalamic volumes in the patients with RLS were also lower than those in healthy controls (0.431 vs. 0.447%, *p* = 0.006; 0.478 vs. 0.502%, *p* = 0.034; respectively). Additionally, the right pulvinar medial, right pulvinar inferior, left lateral posterior, left ventral posterolateral, left ventro-medial, left medial ventral, left medial geniculate, left pulvinar medial, and left pulvinar inferior nuclei volumes in the patients with RLS were lower than those in healthy controls (0.065 vs. 0.070%, *p* = 0.031; 0.013 vs. 0.023%, *p* = 0.012; 0.009 vs. 0.009%, *p* = 0.014; 0.061 vs. 0.065%, *p* = 0.012; 0.015 vs. 0.017%, *p* = 0.012; 0.001 vs. 0.001%, *p* = 0.010; 0.078 vs. 0.085%, *p* = 0.012; 0.079 vs. 0.085%, *p* = 0.016; 0.018 vs. 0.020%, *p* = 0.014; respectively).

In the comparison of the RLS patients with drug-treated state and healthy controls, the right and left whole thalamic volumes in the patients with RLS were not different from those in healthy controls. Furthermore, any of the thalamic nuclei volumes were not different between the groups.

### Intrinsic thalamic network

**Figure 1:**
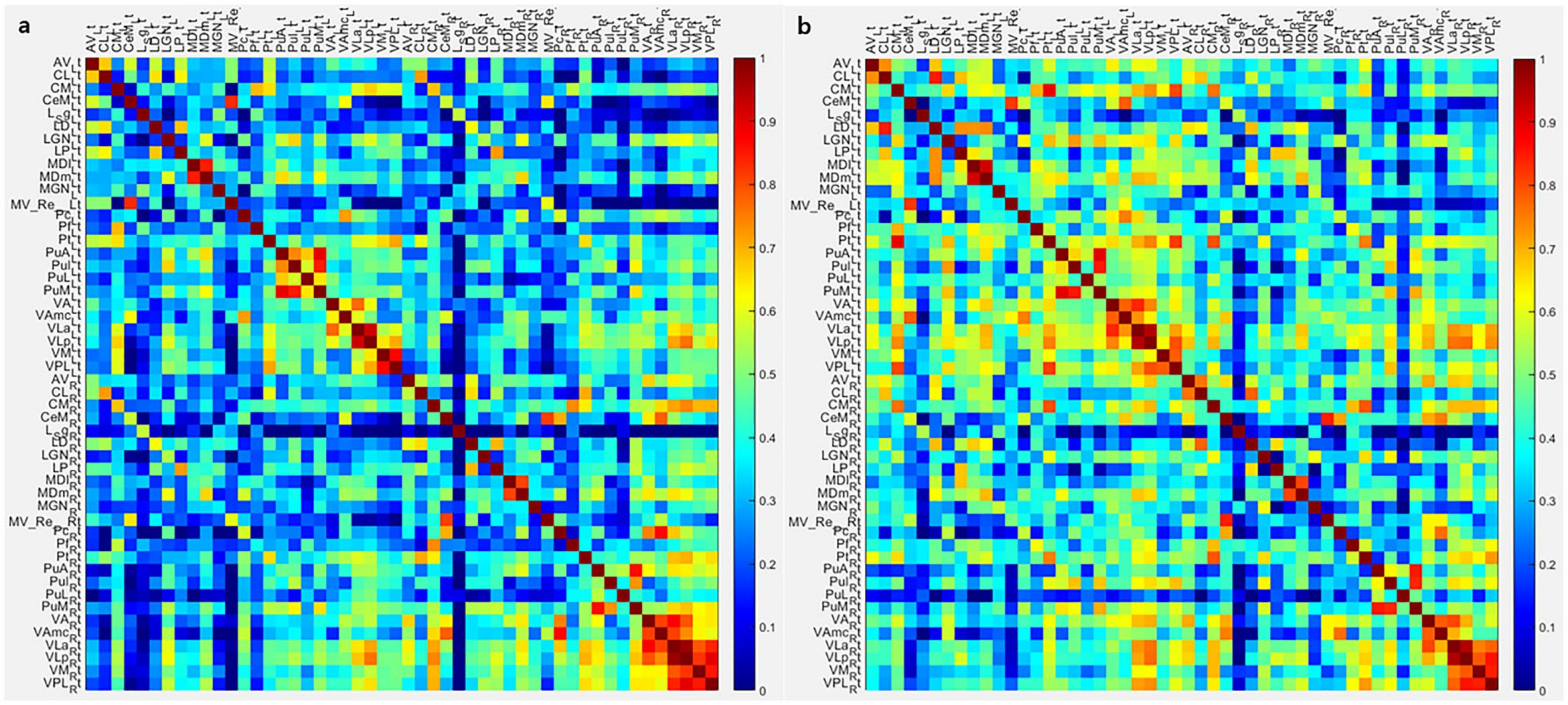
Difference in the intrinsic thalamic network between patients with restless legs syndrome (RLS) and healthy controls. The figures show the connectivity matrix for the intrinsic thalamic network in the patients with RLS (**a**) and healthy controls (**b**).

There was a significant difference in intrinsic thalamic network between patients with RLS and healthy controls (Table 3) (Fig. 1). The assortative coefficient in patients with RLS was higher than that in healthy controls (0.0318 vs. − 0.0358, *p* = 0.048). The other network measures, including characteristic path length, mean clustering coefficient, and small-worldness index were not different between the groups.

**Table 3:** Differences in intrinsic thalamic network between patients with restless legs syndrome and healthy controls.

	Patients with RLS	Healthy controls	CI lower	CI upper	Difference	*p* value	Adjusted* p* value
Characteristic path length	3.2189	2.8292	− 0.6920	0.6864	− 0.3897	0.188	0.246
Mean clustering coefficient	0.3043	0.3458	− 0.1043	0.0967	0.0415	0.246	0.246
Assortative coefficient	0.0318	− 0.0358	− 0.0541	0.0548	− 0.0677	0.012	0.048
Small-worldness index	0.9031	0.9436	− 0.0422	0.0358	0.0406	0.029	0.058

	RLS patients with drug-naïve state	Healthy controls	CI lower	CI upper	Difference	*p* value	Adjusted *p* value
Characteristic path length	3.1210	2.8292	− 0.6513	0.5148	− 0.2918	0.224	0.224
Mean clustering coefficient	0.2964	0.3458	− 0.1897	0.0847	0.0494	0.219	0.224
Assortative coefficient	0.0374	− 0.0358	− 0.0322	0.0501	− 0.0732	0.010	0.040
Small-worldness index	0.9200	0.9436	− 0.0333	0.0148	0.0236	0.032	0.064

	RLS Patients with drug-treated state	Healthy controls	CI lower	CI upper	Difference	*p* value	Adjusted *p* value
Characteristic path length	3.0323	2.8292	− 0.7968	0.9016	− 0.2031	0.375	0.375
Mean clustering coefficient	0.2876	0.3458	− 0.1773	0.1451	0.0582	0.361	0.375
Assortative coefficient	0.0814	− 0.0358	− 0.1113	0.0592	− 0.1172	0.046	0.178
Small-worldness index	0.9013	0.9436	− 0.0346	0.0513	0.0423	0.089	0.178

*RLS* Restless legs syndrome, *CI* 95% confidence interval of the difference between the groups.

In the comparison of the RLS patients with drug-naïve state and healthy controls, the assortative coefficient in patients with RLS was also higher than that in healthy controls (0.0374 vs. − 0.0358, *p* = 0.040). The other network measures were not different between the groups.

In the comparison of the RLS patients with drug-treated state and healthy controls, the intrinsic thalamic network between the patients with RLS and healthy controls was not different.

### Correlation analysis between thalamic nuclei volumes and RLS severity, Pittsburgh sleep quality index, insomnia severity index, hospital anxiety scale and hospital depression scale

No individual thalamic nuclei volumes were significantly correlated with RLS severity (Supplementary Table 1). However, the right thalamic volume was correlated with the Insomnia severity index (r =  − 0.281, *p* = 0.038).

## Discussion

In this study, we newly found that (1) patients with RLS had decreased whole thalamic volumes and some thalamic nuclei volumes, especially the pulvinar inferior, ventral posterolateral, and medial ventral nuclei, compared to healthy controls; (2) there were alterations of the intrinsic thalamic network in patients with RLS; (3) these thalamic alterations were consistently observed even in RLS patients with drug-naïve state; and (4) the whole thalamic and thalamic nuclei volumes as well as the intrinsic thalamic network were not different between RLS patients with drug-treated state and healthy control. These changes might be related to RLS pathophysiology and suggest the pivotal role of the thalamus in RLS symptoms.

The thalamus serves a variety of functions, but it is most commonly thought to act as a hub for the sensory system, relaying information between various subcortical areas and the cerebral cortex^18^. The thalamus receives and transmits sensory signals to the associated primary cortex area. Furthermore, the thalamus has traditionally been thought of as simply a “relay” for signals to the cerebral cortex^18^. However, recent research indicates that the thalamus is more selective and performs a higher order relay function, which is a part of the cortico-thalamo-cortical route of communication^19^. In this study, we found that patients with RLS had lower whole thalamic volumes in both sides than healthy controls. The abnormal sensory processing by thalamic abnormalities reflected with decreased thalamic volumes might lead to sensory symptoms in patients with RLS. These findings are consistent with our previous study discussion of the role of “resting keeper” in the thalamus^20,21^, in which the thalamus could suppress symptoms during a resting state and play an important role in managing RLS symptoms.

Of the several thalamic nuclei, the volumes of the pulvinar inferior, ventral posterolateral, and medial ventral nuclei in patients with RLS were significantly lower than those in healthy controls. The pulvinar inferior thalamic nucleus has widespread connections with the visual cortex, and lesions of the pulvinar region result in visual deficits^22^. In addition, ischemic stroke to the pulvinar nucleus has been linked to the development of chronic pain^22^. The ventral posterolateral thalamic nucleus receives sensory information for the body from the spinothalamic tract and the posterior column-medial pathway’s lemniscus. This sensory information is then projected to the primary sensory cortex^23^. Thus, the decreased volumes in the pulvinar inferior and ventral posterolateral nuclei are related with the main sensory symptoms of RLS. Furthermore, RLS is a sleep related movement disorder characterized by a compulsive urge to move the legs (ie, akathisia). In addition, involuntary movements of the legs, known as periodic limb movements, are frequently present. These sensorimotor symptoms can be associated with decreased volume in the medial ventral thalamic nucleus, which receives afferents from the basal ganglia and communicates information to the cerebral cortex, including motor areas^24^. Thus, the medial ventral thalamic nucleus is associated with at least some aspects of motor function as well as other cortical functions^24^.

We also demonstrated that there were alterations of the intrinsic thalamic network in patients with RLS. Although this is the first study to analyze the intrinsic thalamic network using the covariance analysis method by applying the thalamic nuclei volume-based graph theory, thalamic network alteration has been proven in previous studies using resting state functional MRI (rs-fMRI). Ku et al. enrolled patients with RLS and healthy controls who had undergone rs-fMRI^25^. They discovered that patients with RLS had decreased thalamic connectivity to the parahippocampal, precentral, and lingual gyri, but increased connectivity to the superior temporal, middle temporal, and medial frontal gyri. Additionally, the severity of RLS was negatively correlated with the thalamic-parahippocampal gyrus connection^25^. This finding that RLS is a disorder of the thalamic network was later confirmed in another follow-up study^26^. The study used rs-fMRI on patients with RLS and healthy controls, and assessed functional connectivity changes in the subjects using a seed-based technique utilizing the bilateral ventral and posterolateral thalamic nuclei, and decreased functional connectivity was observed in the bilateral lingual gyri^26^. Our present study revealed that the assortative coefficient in patients with RLS was higher than that in healthy controls. Interestingly, patients with RLS had positive values of assortative coefficients, which is called an assortative network, whereas healthy controls had negative value of assortative coefficient, defined as a disassortative network^27,28^. In an assortative network, a node with many links, that is a hub, is clustered with hubs, whereas nodes with few links are grouped with similar nodes with few links^27,28^. However, in a disassortative network, nodes with many links and nodes with few links are connected. The assortative coefficient quantifies assortativity, or the proclivity of nodes sharing similar properties to connect. Numerous technological and biological networks serve as models for a disassortative network^29^. Additionally, we discovered that healthy controls had a disassortative intrinsic thalamic network in this study. However, in patients with RLS, the intrinsic thalamic network was assortative, which appears to be more difficult to synchronize than a disassortative network^30^. We could assume that less synchronization of the thalamus is associated with dysfunction of the thalamus.

In this study, we firstly investigated the alterations of thalamic nuclei volumes and intrinsic thalamic network in patients with RLS compared to healthy controls, and we enrolled a relatively large sample size of participants. However, there were several limitations. First, less than one-third of the patients with RLS were already taking medication to relieve RLS symptoms prior to MRI scanning. These medications may have affected the volume of the thalamic nuclei. Thus, we additionally analyzed the thalamic changes in RLS patients with drug-naïve state, and we confirmed the alterations of thalamic nuclei volumes and intrinsic thalamic network compared to healthy controls. The whole thalamic and thalamic nuclei volumes as well as the intrinsic thalamic network were not different between RLS patients with drug-treated state and healthy control, which could suggest that thalamic function could return to normal condition by RLS treatment. However, since this study had a cross-sectional design, it was not possible to prove it, and this assumption could be confirmed through the further study with design of longitudinal follow-up MRI scan before and after RLS treatment. Second, our results could not be generalized because the patients were enrolled at a sleep specialist center in one tertiary hospital. Third, because this study was conducted by cross-sectional design, it is difficult to know the causal relationship. Fourth, we could not analyze thalamic changes according to which leg had the dominant RLS symptoms. Lastly, although we measured thalamic nuclei volumes by structural imaging, it is difficult to rule out the possibility that the results may vary depending on the time of MRI scan, because RLS symptoms are more severe at night. In the future, further longitudinal studies with multicenter considering the time of MRI scanning are needed to confirm our findings.

In this study, we demonstrate the alterations of thalamic nuclei volumes and intrinsic thalamic network in patients with RLS compared to healthy controls. These changes might be related with RLS pathophysiology and suggest the pivotal role of the thalamus in RLS symptoms.

## Methods

### Participants: patients with RLS and healthy controls

Seventy-one patients with primary RLS and 55 healthy controls were recruited at a sleep center in a tertiary hospital. This study was performed in accordance with the ethical standards laid down in the 1964 Declaration of Helsinki and its later amendments or comparable ethical standards. The study was approved by the institutional review board of the Keimyung University Dongsan Hospital. Informed consent was obtained from all participating subjects. All methods were carried out in accordance with relevant guidelines and regulations. We enrolled patients with RLS who met the following criteria: (1) primary RLS diagnosed by a certified Korean neurologist (YWC) who is an expert in RLS using the International RLS/WED Study Group (IRLSSG) criteria^1^; (2) no structural lesions on the brain MRI; and (3) no other medical or neurological conditions, except RLS. Patients with secondary RLS due to iron deficiency anemia, pregnancy, chronic kidney disease, or peripheral neuropathy were excluded from the study. Additionally, we recruited healthy subjects as a control group, whose age and gender were comparable to those of the RLS patients. The healthy controls all answered “no” to the RLS diagnostic questionnaire’s initial questions; they had normal brain MRI scans and were free of any medical or neurological conditions.

The International RLS scale was used to assess the severity of RLS symptoms in the study group^31,32^. Additionally, the following scales were assessed with validated Korean-language versions of sleep questionnaires: (1) the Restless Legs Syndrome Quality of Life Questionnaire^32,33^; (2) the Insomnia Severity Index^34^; (3) the Pittsburgh Sleep Quality Index^35^; and (4) the Hospital Anxiety and Depression Scale^36^.

### MRI acquisition: T1-wighed imaging

Patients with RLS and the healthy controls underwent brain MRI using a three-tesla MRI scanner equipped with a 32-channel head coil. Three-dimensional T1-weighted images were acquired using a turbo-field echo sequence with the following parameters: TI = 1300 ms; TR/TE = 8.6/3.96 ms; flip angle = 8°; and isotropic voxel size of 1 mm^3^.

### Obtaining individual thalamic nuclei volumes

**Figure 2:**
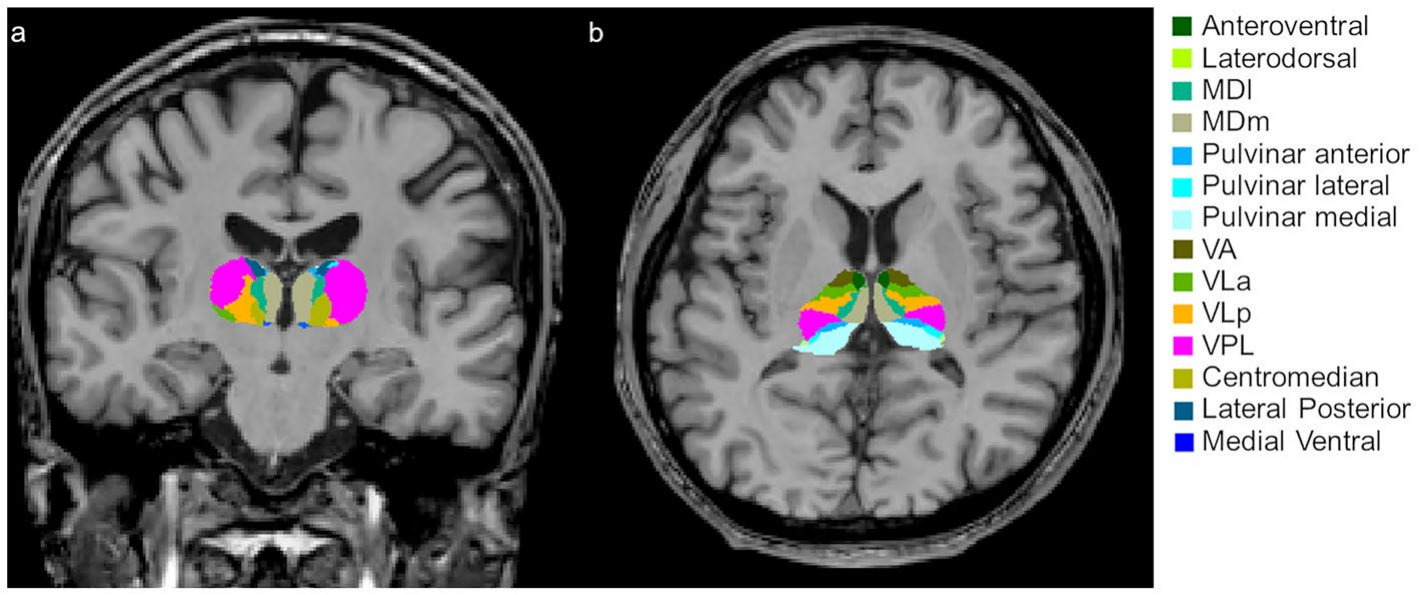
Example of thalamic nuclei segmentation. Segmentations and labels of thalamic nuclei in the coronal (**a**) and axial (**b**) plane generated by FreeSurfer (not all segmentations are shown). The segmentations are overlaid on the T1-weighted scan. This figure is generated from our previous study^17^. *MDl* Mediodorsal lateral parvocellular nucleus, *MDm* Mediodorsal medial magnocellular nucleus, *VLa* Ventral lateral anterior nucleus, *VLp* Ventral lateral posterior nucleus, *VPL* Ventral posterolateral nucleus, *VA* Ventral anterior nucleus.

We obtained whole thalamic volumes and individual thalamic nuclei volumes using the FreeSurfer program (version 7.0). We used the “recon-all” and “segmentThalamicNuclei.sh” functions to automatically obtain the right and left whole thalamic volumes and individual thalamic nuclei volume, respectively^14^. An example of segmentation of thalamic nuclei is demonstrated in Fig. 2. The individual thalamic nuclei included:

- Right and left anteroventral nuclei in the anterior group.
- Right and left laterodorsal and lateral posterior nuclei in the lateral group.
- Right and left ventral anterior, ventral anterior magnocellular, ventral lateral anterior, ventral lateral posterior, ventral posterolateral, and ventromedial nuclei in the ventral group.
- Right and left central medial, central lateral, paracentral, centromedian, and parafascicular nuclei in the intralaminar group.
- Right and left paratenial, medial ventral, mediodorsal medial magnocellular, and mediodorsal lateral parvocellular nuclei in the medial group.
- Right and left lateral geniculate, medial geniculate, suprageniculate, pulvinar anterior, pulvinar medial, pulvinar lateral, pulvinar inferior nuclei in the posterior group.

Then, we corrected the whole thalamic volumes and individual thalamic nuclei volumes by the total intracranial volume. Finally, we investigated the differences in whole thalamic volumes and individual thalamic nuclei volumes between patients with RLS and the healthy controls. In addition, we analyzed the differences in whole thalamic volumes and individual thalamic nuclei volumes between RLS patients with drug-naïve state and the healthy controls, and between RLS patients with drug-treated state and the healthy controls.

### Calculation of intrinsic thalamic network using graph theory and correlation analysis

We previously described the detailed procedure to compute the intrinsic thalamic network using graph theoretical analysis in the BRAPH version 1.0 program^15–17,37^. Graph theory was used to analyze the network’s nodes and edges. We defined nodes as the individual thalamic nuclei volumes, and edges as the partial correlation between the individual thalamic nuclei volumes, while accounting for the effects of age and sex. We created a weighted connectivity matrix from nodes and edges in patients with RLS and healthy controls. Binary links denote the presence or absence of connections, whereas weighted links contain information about connection strength with respective magnitudes of correlational interactions. We subsequently calculated network measures, including characteristic path length, mean clustering coefficient, assortative coefficient, and small-worldness index from the connectivity matrix. The characteristic path length represents the shortest path length between the nodes, which is associated with global integration of the network. The mean clustering coefficient is a measure for the tendency of network elements to form local clusters, and the assortative coefficient is the values for degree of connections between nodes with similar degrees. The small-worldness index describes the balance between local connectedness and global integration in the network^38,39^. These are the most commonly used measures to analyze network topology in graph theory^38,39^. We then analyzed the differences in the network measures between patients with RLS and healthy controls, between RLS patients with drug-naïve state and healthy controls, and between RLS patients with drug-treated state and the healthy controls. We also performed a correlation analysis between individual thalamic nuclei volumes and the RLS severity, the Pittsburgh Sleep Quality Index, the Insomnia Severity Index, and the Hospital Anxiety and Depression Scale in patients with RLS.

### Statistical analysis

Categorical variables were expressed as frequency with percentages, while continuous variables were expressed as mean value with standard deviation (SD) or median value with interquartile range, depending on the normal distribution. In the comparison of the clinical characteristics, the Chi-square test was used to analyze categorical variables, and the independent samples t-test or Mann–Whitney test was used to analyze continuous variables according to the normal distribution. In the comparison of the whole thalamic volumes and thalamic nuclei volumes between the groups, we used an analysis of covariance (ANCOVA) with covariates of age and sex. Pearson’s correlation analysis was used for correlation analysis. We compared network measures using nonparametric permutation tests with 1,000 permutations because we could obtain network measures at the group level. Permutation tests were conducted with the BRAPH program. Multiple false discovery rate corrections (Benjamini–Hochberg procedure)^40^ were used in the comparisons of differences for whole thalamic volumes, individual thalamic nuclei volumes, and network measures between the groups, and in the correlation analysis. MedCalc® Statistical Software version 20.014 (MedCalc Software Ltd, Ostend, Belgium; https://www.medcalc.org; 2021) was used to conduct the statistical analyses. The adjusted *p* value was then displayed. Statistical significance was defined as *p* values less than 0.05.

## Supplementary Information

Supplementary Information.

## Data Availability

All data generated or analyzed during this study are included in this published article (and its Supplementary Information files).
