# Supplementary material for: Alterations of the thalamic nuclei volumes and intrinsic thalamic network in patients with restless legs syndrome
Source: Sci Rep. 2023 Mar 17;13:4415. doi: 10.1038/s41598-023-31606-8 (PMC10023689; doi:10.1038/s41598-023-31606-8)
Supplement: Supplementary file 1 — Supplementary Information. [file 41598_2023_31606_MOESM1_ESM.pdf]

Alterations of the thalamic nuclei volumes and intrinsic thalamic network in patients with restless legs syndrome

Kang Min Park[1,*], Keun Tae Kim[2,*], Dong Ah Lee[1], Yong Won Cho[2]

[1]*Department of Neurology, Haeundae Paik Hospital, Inje University College of Medicine, Busan, Korea*

[2]*Department of Neurology, Keimyung University School of Medicine, Daegu, Korea*

Supplementary Table 1. The results of correlation analysis between thalamic nuclei volumes and restless legs syndrome (RLS) severity, Pittsburgh sleep quality index, Insomnia severity index, Hospital anxiety scale and Hospital depression scale in patients with RLS

		RLS severity	PSQI	ISI	HAS	HDS
	r	-0.141	-0.209	-0.281	-0.189	-0.006
Whole right thalamus	*p*-value	0.241	0.081	0.019	0.117	0.960
	Adjusted *p*-value	0.435	0.081	0.038	0.233	0.960
Right thalamic nucleus						
	r	-0.245	-0.193	-0.279	-0.287	-0.208
anteroventral	*p*-value	0.039	0.107	0.020	0.016	0.084
	Adjusted *p*-value	0.733	0.553	0.201	0.243	0.681
	r	-0.201	-0.193	-0.257	-0.231	-0.206
central medial	*p*-value	0.093	0.107	0.033	0.054	0.087
	Adjusted *p*-value	0.891	0.553	0.205	0.494	0.681
	r	-0.135	-0.102	-0.195	-0.127	-0.164
central lateral	*p*-value	0.261	0.397	0.108	0.295	0.175
	Adjusted *p*-value	0.891	0.873	0.415	0.760	0.681
	r	-0.047	-0.081	-0.061	-0.108	-0.017
centromedian	*p*-value	0.700	0.501	0.617	0.374	0.891
	Adjusted *p*-value	0.974	0.873	0.791	0.760	0.989
	r	0.012	-0.048	-0.083	0.090	0.028
suprageniculate	*p*-value	0.924	0.692	0.496	0.460	0.818
	Adjusted *p*-value	0.996	0.873	0.730	0.767	0.989
	r	-0.137	-0.069	-0.148	-0.284	-0.175
laterodorsal	*p*-value	0.256	0.567	0.225	0.017	0.148
	Adjusted *p*-value	0.891	0.873	0.550	0.243	0.681
	r	-0.082	-0.196	-0.159	-0.173	0.077
lateral geniculate	*p*-value	0.497	0.102	0.191	0.153	0.529
	Adjusted *p*-value	0.974	0.553	0.550	0.747	0.911
	r	-0.031	0.010	-0.039	-0.147	-0.124
lateral posterior	*p*-value	0.800	0.935	0.750	0.224	0.307
	Adjusted *p*-value	0.996	0.972	0.853	0.747	0.769
	r	-0.001	-0.180	-0.348	-0.177	-0.003
mediodorsal lateral parvocellular	*p*-value	0.996	0.132	0.003	0.143	0.978
	Adjusted *p*-value	0.996	0.600	0.067	0.747	0.989
	r	-0.110	-0.230	-0.370	-0.140	0.033
mediodorsal medial magnocellular	*p*-value	0.363	0.053	0.002	0.249	0.787
	Adjusted *p*-value	0.974	0.553	0.067	0.747	0.989
	r	-0.043	-0.191	-0.261	-0.057	-0.153
medial geniculate	*p*-value	0.721	0.111	0.030	0.638	0.207
	Adjusted *p*-value	0.974	0.553	0.205	0.905	0.681
	r	-0.155	-0.101	-0.218	-0.166	-0.146
medial ventral	*p*-value	0.196	0.401	0.072	0.169	0.229
	Adjusted *p*-value	0.891	0.873	0.327	0.747	0.681
	r	-0.051	-0.234	-0.210	-0.043	0.096
paracentral	*p*-value	0.672	0.049	0.083	0.724	0.428
	Adjusted *p*-value	0.974	0.553	0.347	0.905	0.851
	r	0.072	-0.059	-0.069	-0.157	-0.084
parafascicular	*p*-value	0.548	0.626	0.574	0.194	0.490
	Adjusted *p*-value	0.974	0.873	0.763	0.747	0.875

	r	-0.031	-0.138	-0.226	-0.020	0.032
paratenial	*p*-value	0.795	0.250	0.062	0.871	0.796
	Adjusted *p*-value	0.996	0.804	0.309	0.957	0.989
	r	-0.165	-0.197	-0.290	-0.227	-0.103
pulvinar anterior	*p*-value	0.168	0.099	0.016	0.059	0.398
	Adjusted *p*-value	0.891	0.553	0.196	0.494	0.829
	r	-0.129	-0.247	-0.270	-0.134	0.010
pulvinar inferior	*p*-value	0.285	0.038	0.025	0.269	0.934
	Adjusted *p*-value	0.891	0.553	0.205	0.747	0.989
	r	-0.249	-0.119	-0.171	-0.292	-0.171
pulvinar lateral	*p*-value	0.037	0.322	0.161	0.014	0.156
	Adjusted *p*-value	0.733	0.805	0.550	0.243	0.681
	r	-0.115	-0.157	-0.251	-0.158	-0.068
pulvinar medial	*p*-value	0.341	0.190	0.037	0.190	0.577
	Adjusted *p*-value	0.974	0.787	0.207	0.747	0.962
	r	-0.240	-0.316	-0.342	-0.113	0.012
ventral anterior	*p*-value	0.044	0.007	0.004	0.351	0.924
	Adjusted *p*-value	0.733	0.360	0.067	0.760	0.989
	r	-0.090	-0.127	-0.099	-0.097	0.057
ventral anterior magnocelluar	*p*-value	0.455	0.291	0.421	0.427	0.641
	Adjusted *p*-value	0.974	0.804	0.678	0.767	0.989
	r	-0.137	-0.127	-0.068	-0.044	0.131
ventral lateral anterior	*p*-value	0.255	0.292	0.580	0.717	0.281
	Adjusted *p*-value	0.891	0.804	0.763	0.905	0.739
	r	-0.064	-0.040	0.001	-0.079	0.154
ventral lateral posterior	*p*-value	0.597	0.738	0.996	0.513	0.205
	Adjusted *p*-value	0.974	0.873	0.996	0.828	0.681
	r	0.064	0.012	0.027	-0.018	0.111
ventromedial	*p*-value	0.597	0.921	0.825	0.884	0.359
	Adjusted *p*-value	0.974	0.972	0.888	0.957	0.822
	r	-0.020	-0.063	-0.083	-0.064	0.044
ventral posterolateral	*p*-value	0.870	0.604	0.496	0.599	0.719
	Adjusted *p*-value	0.996	0.873	0.730	0.881	0.989
	r	-0.094	-0.104	-0.135	-0.059	0.176
Whole left thalamus	*p*-value	0.435	0.390	0.269	0.628	0.145
	Adjusted *p*-value	0.435	0.390	0.269	0.628	0.290
Left thalamic nucleus						
	r	-0.147	-0.039	-0.077	-0.048	0.107
anteroventral	*p*-value	0.220	0.745	0.529	0.690	0.378
	Adjusted *p*-value	0.891	0.873	0.756	0.905	0.822
	r	-0.007	-0.046	-0.045	-0.090	-0.008
central medial	*p*-value	0.954	0.703	0.713	0.459	0.948
	Adjusted *p*-value	0.996	0.873	0.829	0.767	0.989
	r	-0.050	0.007	-0.127	0.072	0.108
central lateral	*p*-value	0.678	0.953	0.298	0.553	0.375
	Adjusted *p*-value	0.974	0.972	0.583	0.837	0.822
	r	0.012	-0.052	-0.047	-0.137	0.002
centromedian	*p*-value	0.920	0.666	0.703	0.258	0.989
	Adjusted *p*-value	0.996	0.873	0.829	0.747	0.989
	r	-0.024	0.043	-0.001	0.107	-0.005
suprageniculate	*p*-value	0.840	0.725	0.996	0.380	0.966
	Adjusted *p*-value	0.996	0.873	0.996	0.760	0.989
laterodorsal	r	-0.148	-0.036	-0.139	-0.019	0.026

	p-value	0.217	0.768	0.253	0.876	0.830
	Adjusted *p*-value	0.891	0.873	0.567	0.957	0.989
	r	-0.144	-0.195	-0.146	-0.279	-0.016
lateral geniculate	*p*-value	0.231	0.102	0.231	0.019	0.894
	Adjusted *p*-value	0.891	0.553	0.550	0.243	0.989
	r	-0.002	0.066	-0.048	-0.034	0.023
lateral posterior	*p*-value	0.988	0.587	0.694	0.777	0.849
	Adjusted *p*-value	0.996	0.873	0.829	0.948	0.989
	r	0.072	-0.101	-0.058	0.141	0.263
mediodorsal lateral parvocellular	*p*-value	0.550	0.403	0.637	0.243	0.028
	Adjusted *p*-value	0.974	0.873	0.796	0.747	0.681
	r	-0.002	-0.133	-0.123	0.018	0.222
mediodorsal medial magnocellular	*p*-value	0.986	0.268	0.315	0.880	0.065
	Adjusted *p*-value	0.996	0.804	0.583	0.957	0.681
	r	-0.131	-0.075	-0.119	0.012	-0.018
medial geniculate	*p*-value	0.275	0.532	0.330	0.919	0.884
	Adjusted *p*-value	0.891	0.873	0.589	0.957	0.989
	r	-0.017	-0.044	-0.074	-0.160	-0.090
medial ventral	*p*-value	0.886	0.715	0.544	0.185	0.459
	Adjusted *p*-value	0.996	0.873	0.756	0.747	0.851
	r	-0.105	-0.074	-0.137	-0.113	-0.031
paracentral	*p*-value	0.382	0.542	0.261	0.352	0.802
	Adjusted *p*-value	0.974	0.873	0.567	0.760	0.989
	r	-0.036	-0.074	-0.094	-0.151	-0.040
parafascicular	*p*-value	0.767	0.542	0.442	0.212	0.744
	Adjusted *p*-value	0.996	0.873	0.691	0.747	0.989
	r	-0.046	-0.067	-0.133	0.001	0.150
paratenial	*p*-value	0.706	0.577	0.276	0.991	0.216
	Adjusted *p*-value	0.974	0.873	0.574	0.998	0.681
	r	-0.045	-0.011	-0.006	0.102	0.234
pulvinar anterior	*p*-value	0.712	0.925	0.958	0.399	0.052
	Adjusted *p*-value	0.974	0.972	0.996	0.767	0.681
	r	-0.143	-0.123	-0.106	-0.108	0.046
pulvinar inferior	*p*-value	0.235	0.306	0.387	0.372	0.707
	Adjusted *p*-value	0.891	0.804	0.644	0.760	0.989
	r	-0.132	0.074	0.159	0.048	0.059
pulvinar lateral	*p*-value	0.274	0.538	0.192	0.694	0.625
	Adjusted *p*-value	0.891	0.873	0.550	0.905	0.989
	r	-0.085	-0.098	-0.149	0.000	0.145
pulvinar medial	*p*-value	0.479	0.416	0.223	0.998	0.232
	Adjusted *p*-value	0.974	0.873	0.550	0.998	0.681
	r	-0.071	0.021	-0.026	-0.014	0.158
ventral anterior	*p*-value	0.558	0.865	0.835	0.906	0.192
	Adjusted *p*-value	0.974	0.961	0.888	0.957	0.681
	r	-0.006	-0.004	-0.027	-0.072	0.092
ventral anterior magnocelluar	*p*-value	0.963	0.975	0.825	0.553	0.450
	Adjusted *p*-value	0.996	0.975	0.888	0.837	0.851
	r	-0.047	-0.037	-0.111	-0.091	0.203
ventral lateral anterior	*p*-value	0.699	0.758	0.364	0.456	0.092
	Adjusted *p*-value	0.974	0.873	0.627	0.767	0.681
	r	-0.089	-0.074	-0.125	-0.115	0.168
ventral lateral posterior	*p*-value	0.462	0.540	0.306	0.343	0.166
	Adjusted *p*-value	0.974	0.873	0.583	0.760	0.681

	r	-0.091	-0.152	-0.150	0.029	0.177
ventromedial	*p*-value	0.450	0.205	0.218	0.811	0.143
	Adjusted *p*-value	0.974	0.787	0.550	0.957	0.681
	r	-0.086	-0.142	-0.149	-0.049	0.132
ventral posterolateral	*p*-value	0.476	0.237	0.222	0.689	0.278
	Adjusted *p*-value	0.974	0.804	0.550	0.905	0.739

RLS: restless legs syndrome, PSQI: Pittsburgh sleep quality index, ISI: Insomnia severity index, HAS: Hospital anxiety scale, HDS: Hospital depression scale
